# A Healthy Lifestyle Offsets the Increased Risk of Childhood Obesity Caused by High Birth Weight: Results From a Large-Scale Cross-Sectional Study

**DOI:** 10.3389/fnut.2021.736900

**Published:** 2021-11-10

**Authors:** Zheng-he Wang, Zhi-yong Zou, Yan-hui Dong, Rong-bin Xu, Yi-de Yang, Jun Ma

**Affiliations:** ^1^Department of Epidemiology, School of Public Health, Southern Medical University, Guangzhou, China; ^2^School of Public Health, Institute of Child and Adolescent Health, Peking University, Beijing, China; ^3^Department of Epidemiology and Preventive Medicine, School of Public Health and Preventive Medicine, Monash University, Melbourne, VIC, Australia; ^4^Key Laboratory of Molecular Epidemiology of Hunan Province, School of Medicine, Hunan Normal University, Changsha, China

**Keywords:** birth weight, obesity, preventive medicine, lifestyle, child health

## Abstract

**Objective:** To investigate whether a healthy lifestyle is associated with the lower childhood obesity regardless of birth weight.

**Methods:** Participants were selected from a large-scale cross-sectional study conducted in the seven provinces across China. Birth weight and lifestyle factors were collected through a questionnaire. A weighted healthy lifestyle score was calculated and categorized into favorable, intermediate, and unfavorable lifestyles.

**Results:** A total of 47,768 participants were enrolled in this study. Overall, 16.4% of the participants followed a favorable lifestyle, 62.8% followed an intermediate lifestyle, and 20.8% followed an unfavorable lifestyle. Compared with the participants who were born normal birth weight (NBW), participants who were born high birth weight (HBW) (OR = 1.58; 95% CI 1.48–1.77) and very high birth weight (VHBW) (OR = 1.79; 95% CI: 1.47–2.18) had higher obesity risk, however, the participants who were born low birth weight (LBW) had lower obesity risk (OR = 0.81; 95% CI: 0.68–0.96). Participants with an unfavorable lifestyle were associated with a higher risk of childhood obesity compared with the participants with favorable lifestyle (OR = 1.25; 95%CI: 1.14–1.38). Participants who were born VHBW and with an unfavorable lifestyle had 2.76 times (95% CI: 1.78–4.28) further risk of childhood obesity compared with the participants who were born NBW and with a favorable lifestyle. However, adherence to a favorable lifestyle seems to counteract the elevated risk of childhood obesity by VHBW (OR = 1.37; 95% CI: 0.84–2.24).

**Conclusion:** Both the HBW and unfavorable lifestyle were significantly associated with risk of childhood obesity. Adherence to a favorable lifestyle decreased the risk of childhood obesity among the participants with VHBW. A more longitudinal study is required to repeat the finding to inform tailored prevention programs.

## Introduction

Childhood obesity is a major health problem both in the developed and developing countries (1). In the United States, the prevalence of obesity was 16.9% (95% CI, 14.9–19.2%) in children and adolescents aged 2–19 years (2). Similarly, the prevalence of childhood obesity has substantially increased over the past 30 years in China. According to a series, cross-sectional surveys of the Chinese National Survey on Students' Constitution and Health from 1985 to 2014, overweight and obesity prevalence continually increased from 1.1% in 1985 to 20.4% in 2014 in Chinese school-aged children (3). Childhood obesity has significantly adverse effect on both the physical and psychological health (4). Children with obesity are likely to remain obese even into adulthood and are more likely to develop non-communicable diseases, such as diabetes mellitus and cardiovascular diseases at a younger age (5–8). Our previous study had observed that compared with the children with normal weight, children with overweight (OR = 1.49, 95% CI 1.01, 3.10) and obesity (OR = 1.86, 95% CI 1.12, 3.10) were associated with a higher risk of impaired fasting glycemia (8).

Many factors play an important role in the occurrence of childhood obesity. Both the birth weight and lifestyle factors were associated with the childhood obesity. Studies showed that birth weight was associated with a later risk for obesity. The vast majority of studies observed that the higher birth weight (HBW) had a positive association with the risk of childhood obesity and diabetes, such as studies from 11 European countries (9), the United States (10), Turkey (11), China (12), and Korea (13). There is mounting evidence that children with a favorable lifestyle, such as having a healthy diet, adequate sleeping time, regular physical activity (PA), and appropriate screen time had a lower risk of obesity (14–16). Recently, the studies have combined these lifestyle factors to calculate a lifestyle score to assess the association of lifestyle and health outcomes in children and adolescents, such as cardiometabolic problems and obesity (17). In addition, usually the risk factors do not affect obesity alone, they work together complexly, interacting or having combined effects to impact on the risk of obesity. Therefore, we speculated that it is possible that the increased risk of high birth weight on childhood obesity could be offset by a healthy lifestyle. However, the evidence is still limited.

The purpose of the study is to test the hypothesis that adherence to a healthy lifestyle may offset the increased risk of childhood obesity caused by HBW using the data from a national large-scale cross-sectional study.

## Materials and Methods

This study is based on the data of a large-scale cross-sectional study conducted in the seven provinces across mainland China that received approval from the Ethical Committee of the Peking University (IRB00001052-13034). All the participant students and their parents provided informed consents voluntarily.

### Study Design and Population

The cross-sectional study was performed from September to October 2013, which selected participants aged 6–18 years from the seven provinces across the mainland China, including Tianjin, Shanghai, Liaoning, Chongqing, Hunan, Ningxia, and Guangdong. This survey used a standardized and uniform protocol in all the selected schools across the selected provinces. A multistage cluster random sampling method was used to the selected participants. Briefly, first, three to four districts from each province were randomly selected. Second, 12–16 schools were chosen from each district. Third, two to three classes per grade were selected randomly, after excluding students with the serious organic diseases or those who refused to sign informed consent, all the students aged 6–18 years from the selected classes were selected as the participants in the survey. A staff member who was not involved in the survey performed the randomization process. Details of the design have been described carefully in a previous study (18). In this study, we enrolled 47,768 students aged 6–18 years with the gestational age ≥37 weeks into the final analysis.

### Data Collection and Questionnaire Survey

Data on the students aged 6–18 years were collected using the standard questionnaires that were filled in by the participants and one of their parents. Data of the intake frequency of common eaten foods in the Chinese children, sleep duration, PA, and screen time were reported by the participants. However, the demographic information, feeding situation, parental education attainment, the delivery method, birth weight, gestation age, family history of the diseases, and body mass index of mother were reported by their parents.

Dietary consumption, including fruits, vegetables, meats, dairy, sugar-sweetened beverages, desserts, and fried foods were reported by the participants. They reported the frequency (day) and amount (servings) over the past a week (7 days). The average daily intake of the single food was calculated by (day × amount in each of those days) ÷ 7 (19). PA was collected using the International Physical Activity Questionnaire Short Form (IPAQ-SF) (20).

### Birth Weight Collection and Categorization

Data of birth weight and gestational age were collected using a standard parent/guardian questionnaire. Most of the parents/guardians (70.9%) reported birth weight and gestational age of their child according to the birth certificate that was made by the hospital after birth. For those who do not have the birth certificate, we required parents/guardians reported birth weight and gestational age of their child based on the measurement by themselves. Moreover, we repeated the questionnaire survey after 6 months later, and found that the difference of the birth weight and gestational age between these two surveys was <10%. After excluding the participants with gestational age <37 weeks, all the participants were categorized into low birth weight (LBW) (birth weight <2,500 g), normal birth weight (NBW) (birth weight: 2,500–3,999 g), high birth weight (HBW) (birth weight: 4,000–4,499 g), and very high birth weight (VHBW) (birth weight ≥ 4,500 g) according to their birth weight (21).

### Healthy Lifestyle Score

A healthy lifestyle score was calculated using four well-established obesity risk factors (diet, PA, screen time, and sleep duration) assessed at the baseline survey through a questionnaire (22–24). Participants scored one point for each of the four healthy behaviors defined according to the national recommendations for the children and adolescents listed later. Regular PA was defined as if the participants meeting the recommendation of the American Heart Association, which recommends at least 150 min of the moderate PA per week or 75 min of vigorous PA per week, or an equivalent combination (25). Screen time was defined as screen time <2 h per day and screen time ≥2 h per day based on the sum of the time spent watching TV and the tablet or smartphone. Sleep duration was defined as an adequate sleep and inadequate sleep according to the recommendation of the Canadian sedentary behavior guidelines for the children and youth (26). Healthy diet was based on the seven common eaten foods (including fruit, vegetable, sugar-sweetened beverage, meat, milk, dessert, and fry foods) that have been documented associated with childhood obesity (27–31). A healthy diet was defined if the students whose consumption of at least four of seven common eaten foods following recommendations on the Dietary Guidelines for the Chinese Residents (2016) (32). The lifestyle index scores ranged from zero to four, with the higher score indicating healthier lifestyles, and were further categorized as unfavorable lifestyles (zero or one healthy lifestyle factors), intermediate lifestyles (two healthy lifestyle factors), and favorable lifestyles (three or four healthy lifestyle factors). A weighted standardized healthy lifestyle score was then derived based on β coefficients of each lifestyle factor in the binary logistic regression model with all the four lifestyles factors and the adjustment for age, sex, region, and ethnicity. The original binary lifestyle variables were multiplied by the β coefficients, summed, divided by the sum of the β coefficients, and multiplied by 100. The weighted standardized lifestyle score was categorized as favorable, intermediate, and unfavorable based on the distribution of the unweight lifestyle score (33).

### Obesity Definition

The Chinese BMI percentile criterion for screening overweight and obesity in the children and adolescents was used to define the obesity among the students aged 6–18 years (34). Participants with age- and sex-specific BMI ≥ 95th percentile were defined as obesity.

### Anthropological Measurements

Height and weight were measured by a trained team following a standardized procedure. Height was assessed by the portable stadiometer (model TZG, Hengsheng Physical Examination Equipment Co., Ltd. China) to the nearest 0.1 centimeter, with the participants standing straight without shoes and wearing light clothes only. Weight was assessed by the lever-type weight scale (model RGT-140, Hengsheng Physical Examination Equipment Co., Ltd. China) to the nearest 0.1 kg. Both the height and weight were measured twice, and the average value was calculated. About 5% of the participants would be rechecked per day for both height and weight. If the error exceeds 10%, all the participants would be measured again.

### Statistical Analysis

Normally distributed continuous variables were shown as mean and SD, and categorical variables were shown as percentages. The Student's *t*-test was used to compare the mean age between subjects with obesity and subjects with non-obesity. The chi-squared test was used to compare the difference of distribution of the categorical variables between subjects with the obesity and subjects with non-obesity. The binary logistic regression models were used to examine the association of the birth weight, lifestyle, and the combination of birth weight and lifestyle categories (nine categories with NBW and favorable lifestyle as the reference group) with the risk of obesity in children and adolescents aged 6–18 years. All the logistic regression models were adjusted for age, sex, region, and ethnicity. All the analyses were performed using IBM SPSS Statistics version 25.0 and *P* < 0.05 with two-sided were considered to be statistically significant.

## Results

Basic characteristics of the subjects are provided in Table 1. A total of 47,768 subjects were enrolled into this study, including 5,426 (11.4%) subjects with obesity and 42,342 (88.6%) subjects with non-obesity. The mean age was younger in the subjects with obesity compared with those in the subjects with non-obesity (10.0 vs. 10.7 years; *P* < 0.001). The distribution of sex, region, healthy diet, regular screen time, the number of healthy lifestyle factors, birth weight category, and obesity of the mother was different between obesity and non-obesity (*P* < 0.05). However, the proportion of ethnicity, regular PA, and adequate sleep time was similar between obesity and non-obesity (*P* > 0.05). Most participants engaged in either one (25.7%) or two (45.4%) of four healthy lifestyle factors, only 2.7% of participants engaged in the four healthy lifestyle factors. For the weighted lifestyle score, 20.8% were categorized as following an unfavorable lifestyle (scores ranging from 0 to 41), 62.8% followed an intermediate lifestyle (scores ranging from 42 to 87), and 16.4% followed a favorable lifestyle (scored ranging from 88 to 100). Of 3.4% participants who were born LBW, 86.5% of participants who were born NBW, 8.7% of the participants who were born HBW, and 1.4% of the participants who were born VHBW.

**Table 1:** Basic characteristics of study population in a study of the association between birth weight and lifestyle with obesity in the children and adolescents.

**Characteristic**	**No. (%)**			***P-*value**
	**Total (*n* = 47,768)**	**Non-obesity (*n* = 42,342)**	**Obesity (*n* = 5,426)**	
Age, mean (SD), y	10.6 (3.2)	10.7 (3.3)	10.0 (2.9)	<0.001
**Sex**				<0.001
Male	24,142 (50.5)	20,704 (48.9)	3,438 (63.4)	
Female	23,626 (49.5)	21,638 (51.1)	1,988 (36.6)	
**Region**				<0.001
Rural	26,969 (56.5)	24,199 (57.2)	2,770 (51.1)	
Urban	20,799 (43.5)	18,143 (42.8)	2,656 (48.9)	
**Mother obesity**				<0.001
Yes	1,900 (4.0)	1,428 (3.4)	472 (8.7)	
No	45,868 (96.0)	40,914 (96.6)	4,954 (91.3)	
**Ethnicity**				0.826
Han	43,405 (90.9)	38,479 (90.9)	4,926 (90.8)	
Others	4,363 (9.1)	3,863 (9.1)	500 (9.2)	
**Healthy lifestyle factors**				
Healthy diet	14,899 (31.2)	13,316 (31.4)	1,583 (29.2)	0.001
Regular physical activity	27,262 (57.1)	24,157 (57.1)	3,105 (57.2)	0.809
Regular Screening time	34,996 (73.3)	31,135 (73.5)	3,861 (71.2)	<0.001
Adequate sleep	15,629 (32.7)	13,812 (32.6)	1,817 (33.5)	0.200
**No. of healthy lifestyle factors**				0.016
0	1,858 (3.9)	1,628 (3.8)	230 (4.2)	
1	12,275 (25.7)	10,791 (25.5)	1,484 (27.3)	
2	21,688 (45.4)	19,287 (45.6)	2,401 (44.2)	
3	10,653 (22.3)	9,489 (22.4)	1,164 (21.5)	
4	1,294 (2.7)	1,147 (2.7)	147 (2.7)	
**Birth weight**				<0.001
LBW	1,644 (3.4)	146 (2.7)	1,498 (3.5)	
NBW	41,322 (86.5)	4,467 (82.3)	36,855 (87.0)	
HBW	4,137 (8.7)	683 (12.6)	3,454 (8.2)	
VHBW	665 (1.4)	130 (2.4)	535 (1.3)	

*LBW, low birth weight; NBW, normal birth weight; HBW, high birth weight; VHBW, very high birth weight; No., number*.

As shown in Table 2, compared with the participants who were born NBW, participants who were born LBW had lower risk of childhood obesity (OR = 0.81; 95% CI: 0.68–0.96; *P* = 0.017), however, the participants who were born HBW (OR = 1.58; 95% CI: 1.44–1.73; *P* < 0.001) and VHBW (OR = 1.78; 95% CI: 1.46–2.18; *P* < 0.001) had a higher risk of childhood obesity after an adjusted for age, sex, region, mother obesity, and ethnicity. Additional adjustment for the weighted lifestyle factors did not change these associations, indicating that the obesity risk was statistically independent of the lifestyle factors.

**Table 2:** Association between the birth weight category and obesity in children and adolescents.

**Birth weight**	**Model 1^a^**				**Model 2^b^**			
	**NBW (*n* = 41,322)**	**LBW (1,644)**	**HBW (*n* = 4,137)**	**VHBW (*n* = 665)**	**NBW (*n* = 41,322)**	**LBW (1,644)**	**HBW (*n* = 4,137)**	**VHBW (*n* = 665)**
*OR* (95%*CI*)	1 [Reference]	0.81 (0.68–0.96)	1.58 (1.44–1.73)	1.78 (1.46–2.18)	1 [Reference]	0.81 (0.68–0.96)	1.58 (1.48–1.77)	1.79 (1.47–2.18)
*P*-value		0.017	<0.001	<0.001		0.017	<0.001	<0.001
*P*-value for trend		<0.001				<0.001		

*LBW, low birth weight; NBW, normal birth weight; HBW, high birth weight; VHBW, very high birth weight; OR, odds ratio*.

a *Binary logistic regression model adjusted for age, sex, region, mother obesity, and ethnicity*.

b *Binary logistic regression model adjusted for model 1 and weighted lifestyle factors*.

The association between lifestyle and risk of the childhood obesity was presented in Table 3. The risk of childhood obesity was significantly higher (OR = 1.25; 95% CI: 1.14–1.37; *P* < 0.001) in the participants with an unfavorable lifestyle compared with the participants with favorable lifestyle after adjusted for age, sex, region, mother obesity, and ethnicity. Additional adjustment of the birth weight category resulted in an OR of 1.25 (95% CI: 1.14–1.38; *P* < 0.001), consistent with the independence of birth weight and lifestyle risk factors.

**Table 3:** Association between lifestyle and obesity in children and adolescents.

**Healthy lifestyle category**	**Model 1^a^**			**Model 2^b^**		
	**Favorable (*n* = 9,941)**	**Intermediate (*n* = 30,013)**	**Unfavorable (*n* = 7,814)**	**Favorable (*n* = 9,941)**	**Intermediate (*n* = 30,013)**	**Unfavorable (*n* = 7,814)**
*OR* (95%*CI*)	1 [Reference]	1.05 (0.98–1.14)	1.25 (1.14–1.37)	1 [Reference]	1.05 (0.98–1.14)	1.25 (1.14–1.38)
*P*-value		0.171	<0.001		0.166	<0.001
*P*-value for trend	<0.001			<0.001		

*OR, odds ratio*.

a *Binary logistic regression model adjusted for age, sex, region, mother obesity, and ethnicity*.

b *Binary logistic regression model adjusted for model 1 and birth weight category*.

**Figure 1:**
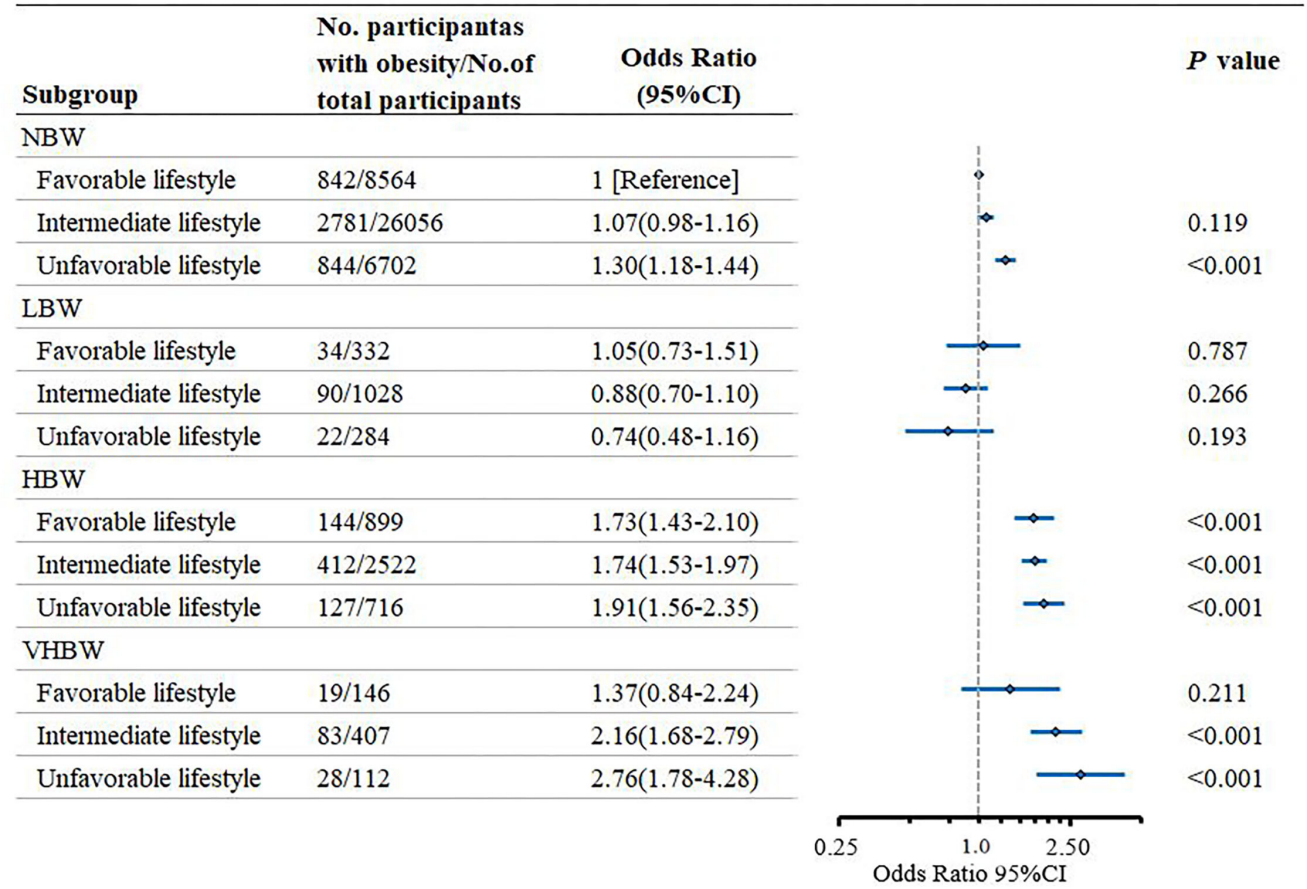
Risk of obesity according to the birth weight and lifestyle in the children and adolescents.

When the birth weight category and lifestyle category were combined there was a monotonic association with increasing birth weight and unhealthy lifestyle (Figure 1). Of the participants who were born VHBW and with an unfavorable lifestyle had a significantly higher obesity risk than the participants who were born NBW and with a favorable lifestyle (OR = 2.76; 95% CI: 1.78–4.28; *P* < 0.001). However, adherence to a favorable lifestyle seems to counteract the elevated risk of childhood obesity by VHBW (OR = 1.37; 95% CI: 0.84–2.24). Consistent association was also observed when compared with the participants who were born NBW and with an unfavorable lifestyle (Supplementary Figure 1). There was no significant interaction between birth weight category and weighted healthy lifestyle category (*P* = 0.273) indicating that the association with lifestyle factors did not vary substantially on the basis of the birth weight.

Table 4 presented the association between lifestyle and childhood obesity risk stratified by the birth weight category. Among the participants who were born VHBW, participants with a favorable lifestyle had a lower risk of obesity than the participants with an unfavorable lifestyle (OR = 0.50; 95% CI: 0.26–0.95; *P* = 0.036). Among the participants who were born NBW, compared with the participants with unfavorable lifestyle, participants with favorable lifestyle also had significantly lower risk of childhood obesity (OR = 0.77; 95% CI: 0.69–0.85; *P* < 0.001).

**Table 4:** Risk of obesity according to a healthy lifestyle category within each birth weight in the children and adolescents.

**Healthy lifestyle category**	**LBW**			**NBW**			**HBW**			**VHBW**		
	**Favorable (*n* = 332)**	**Intermediate (*n* = 1,028)**	**Unfavorable (*n* = 284)**	**Favorable (*n* = 8,564)**	**Intermediate (*n* = 26,056)**	**Unfavorable (*n* = 6,702)**	**Favorable (*n* = 899)**	**Intermediate (*n* = 2,522)**	**Unfavorable (*n* = 716)**	**Favorable (*n* = 146)**	**Intermediate (*n* = 407)**	**Unfavorable (*n* = 122)**
*OR* (95%*CI*)	1.42 (0.81–2.50)	1.18 (0.72–1.92)	1 [Ref.]	0.77 (0.69–0.85)	0.82 (0.76–0.89)	1 [Ref.]	0.90 (0.69–1.18)	0.89 (0.72–1.12)	1 [Ref.]	0.50 (0.26–0.95)	0.76 (0.46–1.25)	1 [Ref.]
*P*-value	0.223	0.508		<0.001	<0.001		0.457	0.325		0.036	0.276	
*P*-value for trend		0.273			<0.001			0.375			0.014	
*P*_interaction_						0.273						

*LBW, low birth weight; NBW, normal birth weight; HBW, high birth weight; VHBW, very high birth weight; Ref., reference; OR, odds ratio*.

## Discussion

Using the data from a large-scale cross-sectional study in the Chinese children and adolescents aged 6–18 years old, this study observed that high birth weight and healthy lifestyle were independently associated with the risk of childhood obesity. Participants with HBW and unfavorable lifestyle had a significantly higher risk of childhood obesity compared with those with NBW and a favorable lifestyle. There was no significant interaction between HBW and healthy lifestyle, and a favorable lifestyle was associated with a lower risk of the childhood obesity regardless of the birth weight.

The increased risk of the HBW on childhood obesity was similar to the previous meta-analysis, which combined data from 162,129 mothers and their children from 37 pregnancy and birth cohort studies from Europe, North America, and Australia, and found that the participants with high birth weight had 0.72 times higher risk of obesity in the late childhood (10–18 years) compared with the participant with NBW (35). Although the majority of the previous studies had observed that the healthy lifestyle factors was negatively associated with the risk of childhood obesity (16, 36, 37), there was no study that examined the association between lifestyle factors and childhood obesity using a combined-weighted lifestyle score. In this study, we combined four lifestyle factors of obesity and calculated a weighted lifestyle score, and found that the participants with an unfavorable lifestyle had 0.26 times higher risk of childhood obesity than those with a favorable lifestyle. They were indicating that a healthy lifestyle may decrease the risk of obesity in childhood.

In contrast, we observed that the low-birth weight was a protective factor of childhood obesity, which was consistent with the previous studies (38–40). This finding suggests that the participants with poor nutritional status during pregnancy might still exhibit these conditions when they reach the school age.

To our knowledge, no previous study had investigated the association of the combined birth weight and lifestyle with the risk of childhood obesity. A previous study examined the association of the combined polygenic risk score and lifestyle with the childhood obesity in 997 children, and found that among children with high-polygenic risk, a healthy lifestyle was associated with an 85% lower risk of obesity compared with an unhealthy lifestyle (OR = 0.15; 95% CI: 0.04–0.59; *P* = 0.007) (41). However, the lifestyle score in their study was calculated as the sum of seven lifestyle factors, including slowness in eating, satiety responsiveness, food responsiveness, screen time, PA, sugar-sweetened beverages consumption, and sleep duration, but not weighted used the β coefficient of each factor with obesity in the logistic regression model. Furthermore, the previous study had limited statistical power due to the limited sample size. Thus, the effect of lifestyle on childhood obesity may be misassessed. Compared with the previous study, this study used a large-scale national sample, and a weighted lifestyle score that investigated the association of HBW and lifestyle with the risk of childhood obesity.

This study found that HBW increased the risk of obesity, and the increased risk might be largely offset by the favorable lifestyle in childhood, especially for those with VHBW. Compared with the participants who were born NBW, participants who were born VHBW increased 79% risk of the childhood obesity. Fortunately, if these participants adherence to a favorable lifestyle in their later life, they might decrease 50% risk of childhood obesity compared with those adherences to an unfavorable lifestyle. These findings indicate that the increased risk of the childhood obesity caused by HBW might be largely offset by a favorable lifestyle in the later life. Furthermore, participants who were born VHBW and adherence to a favorable lifestyle did not significantly increase the risk of childhood obesity compared with the participants who were born NBW and adherence to a favorable lifestyle. This finding furthermore supported that the increased risk of childhood obesity caused by HBW might be largely offset by a favorable lifestyle in their later life. These findings have an important public health implication because individuals who were born HBW have a higher risk of childhood obesity, but the elevated risk could be reduced if they adhere to a favorable lifestyle in their later life.

Despite there was no study examining the association of birth weight and lifestyle with the risk of childhood obesity, a previous study conducted by Qiao et al. explored the joint association of birth weight and PA/sedentary behavior with childhood obesity and found that the moderate and vigorous PA is more important than high birth weight as a correlate of obesity in children (42). It was inconsistent with the findings from this study, we found that the increased risk of childhood obesity caused by an unfavorable lifestyle seems to be less than that caused by high birth weight. We speculated that the differences in population and measurement methods of PA might explain the inconsistent results. In the study by Qiao et al., participants were from 12 countries (Australia, Brazil, Canada, China, Colombia, Finland, India, Kenya, Portugal, South Africa, the United Kingdom, and the United States) and an ActiGraph GT3X+ accelerometer was used to assess the PA level. However, this study only selected participants from the Chinese children and adolescents and IPAQ-short form was used to assess the level of PA.

This study has several limitations. First, the association observed in this study comes from a cross-sectional study that prevents us from making causal inferences. Second, the data of birth weight was retrospectively collected from the parents of participants. Thus, the association in this study might be overestimated or underestimated. However, birth weight was obtained from their birth certificate or the health clinic card. Third, although analyses were adjusted for many variables that potentially caused bias, the unmeasured confounding and reverse causation remains. Four, lifestyle factors were collected using a self-reported questionnaire, which possibly remained recall bias and resulted in the misclassification errors. However, the misclassification errors are likely to make these findings toward the null. Five, other lifestyle or environmental factors might also be associated with the risk of childhood obesity. Six, the association has not been validated in other independent population.

In conclusion, this study used the data from a large-scale cross-sectional study in the Chinese children and adolescents aged 6–18 years, and found that the high birth weight and healthy lifestyle were independently associated with the risk of childhood obesity, and the increased risk caused by the high birth weight might be largely offset by a favorable lifestyle in children. These findings have important implications for the population with high birth weight to adherence to a healthy lifestyle to prevent obesity in their later life.

## Data Availability Statement

The raw data supporting the conclusions of this article will be made available by the authors, without undue reservation.

## Ethics Statement

The studies involving human participants were reviewed and approved by the Ethical Committee of the Peking University. Written informed consent to participate in this study was provided by the participants' legal guardian/next of kin.

## Author Contributions

JM, Z-yZ, and Z-hW were co-investigators and designed the study. Z-hW and Y-hD carried out the initial analysis. R-bX and Y-dY supervised data analysis. Z-hW take full responsibility for the complete work. All the authors were involved in writing the paper and had final approval of the submitted and published versions.

## Funding

This project was supported by the Research Special Fund for Public Welfare Industry of Health (Grant Number 201202010).

## Conflict of Interest

The authors declare that the research was conducted in the absence of any commercial or financial relationships that could be construed as a potential conflict of interest.

## Publisher's Note

All claims expressed in this article are solely those of the authors and do not necessarily represent those of their affiliated organizations, or those of the publisher, the editors and the reviewers. Any product that may be evaluated in this article, or claim that may be made by its manufacturer, is not guaranteed or endorsed by the publisher.
