# Supplementary material for: A Healthy Lifestyle Offsets the Increased Risk of Childhood Obesity Caused by High Birth Weight: Results From a Large-Scale Cross-Sectional Study
Source: Front Nutr. 2021 Nov 10;8:736900. doi: 10.3389/fnut.2021.736900 (PMC8631431; doi:10.3389/fnut.2021.736900)

Supplementary Materials

Figure S1 Risk of obesity according to birthweight and lifestyle in children and adolescents using participants with normal birthweight and unfavorable lifestyle as a reference group.

Subgroup	No. participantas with obesity/No.of total participants	Odds Ratio (95%CI)	*P* value
NBW			
Unfavorable lifestyle	844/6702	1 [Reference]	
Intermediate lifestyle	2781/26056	0.80(0.73-0.86)	<0.001
Favorable lifestyle	842/8564	0.75(0.68-0.83)	<0.001
LBW			
Favorable lifestyle	34/332	0.81(0.56-1.16)	0.248
Intermediate lifestyle	90/1028	0.67(0.54-0.85)	0.001
Unfavorable lifestyle	22/284	0.57(0.37-0.89)	0.013
HBW			
Unfavorable lifestyle	127/716	1.56(1.27-1.92)	<0.001
Intermediate lifestyle	412/2522	1.35(1.19-1.54)	<0.001
Favorable lifestyle	144/899	1.37(1.13-1.7)	0.001
VHBW			
Unfavorable lifestyle	28/112	2.36(1.52-3.65)	<0.001
Intermediate lifestyle	83/407	1.76(1.37-2.27)	<0.001
Favorable lifestyle	19/146	1.09(0.67-1.78)	0.734

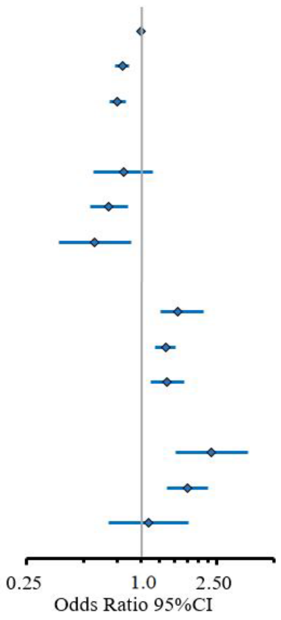

Supplement: Supplementary file 1 [file Data_Sheet_1.PDF]
